# DNAJB9 promotes epithelial-mesenchymal transition in the nasal epithelium of chronic rhinosinusitis by enhancing TRIM22-mediated IκBα degradation

**DOI:** 10.1016/j.isci.2026.116681

**Published:** 2026-07-16

**Authors:** Zhiqiang Zhang, Xinyu Huang, Junhao Tu, Mengyuan Liu, Ying Wu, Xu Zhang, Yun Zhang, Chunping Yang

**Affiliations:** 1 Department of Otorhinolaryngology, Head and Neck Surgery, The Second Affiliated Hospital, Jiangxi Medical College, Nanchang University, Nanchang, Jiangxi Province, China; 2 Jiangxi Department of Radiology, The Second Affiliated Hospital, Jiangxi Medical College, Nanchang University, Nanchang, Jiangxi Province, China

**Keywords:** chronic sinusitis, epithelial-mesenchymal transition, DNAJB9, ubiquitination, NF-κB

## Abstract

This study investigates the functional role of DNAJB9 in the pathogenesis of chronic rhinosinusitis with nasal polyps (CRSwNP). Using quantitative proteomics alongside human tissue analysis and murine models, we found that DNAJB9 is significantly upregulated in CRSwNP nasal epithelium and closely mirrors epithelial-mesenchymal transition (EMT) progression. Functional assays in human epithelial cells demonstrated that DNAJB9 actively drives the EMT phenotype. Mechanistically, DNAJB9 interacts with and stabilizes the E3 ubiquitin ligase TRIM22 by inhibiting its autoubiquitination. This stabilized TRIM22 targets IκBα for proteasomal degradation, leading to constitutive NF-κB pathway activation and subsequent EMT. Ultimately, DNAJB9 acts as an essential driver of tissue remodeling in CRSwNP. These findings highlight the broader clinical significance of the DNAJB9/TRIM22/NF-κB signaling axis, presenting it as a promising therapeutic target for inhibiting nasal polyp formation and associated remodeling.

## Highlights

• DNAJB9 is significantly upregulated in human CRSwNP nasal tissue
• DNAJB9 expression correlates positively with EMT progression
• DNAJB9 drives epithelial-mesenchymal transition in nasal cells
• DNAJB9 activates NF-κB via TRIM22-mediated IκBα degradation

## Graphical abstract

**Figure:**
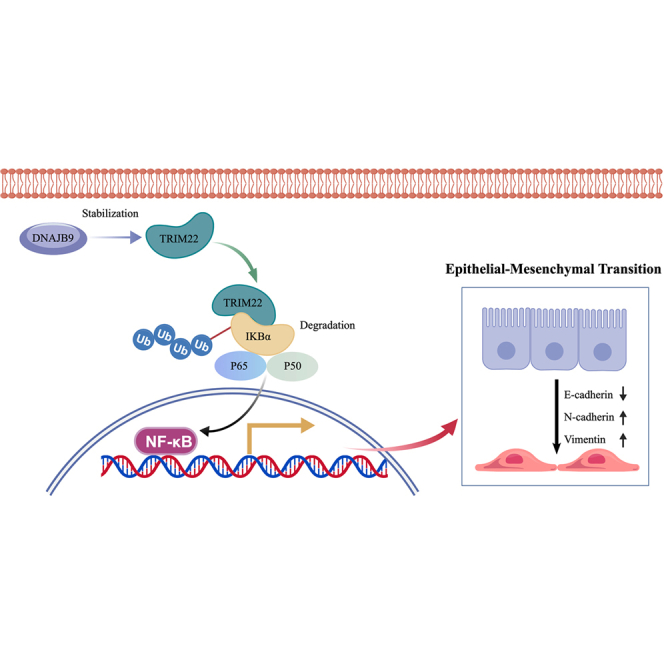

## Teaser

Biological sciences; Cell biology; Molecular biology

## Introduction

Chronic rhinosinusitis (CRS) is a highly prevalent chronic inflammatory disease of the upper respiratory tract with a global prevalence of approximately 10.9%. The disease not only seriously impairs the quality of life of patients but also significantly increases the social medical burden.^1^ During the progression of CRS, prolonged chronic inflammatory stimulation triggers structural and functional abnormalities of the nasal mucosal epithelium, a pathological process known as epithelial remodeling. Of these processes, epithelial-mesenchymal transition (EMT)—the process by which epithelial cells lose polarity and acquire a mesenchymal phenotype—has been identified as a core mechanism underlying epithelial remodeling in CRS.^2^ EMT is found in both CRS with nasal polyps (CRSwNP) and CRS without nasal polyps (CRSsNP), indicating that it plays an important pathological role in different clinical phenotypes.^3^^,^^4^

The histopathological differences between CRSsNP and CRSwNP further suggest that there are different remodeling patterns: CRSsNP is mainly characterized by mucosal fibrosis, basement membrane thickening, and collagen deposition, while CRSwNP is characterized by tissue edema, inflammatory cell infiltration, and epithelial barrier disruption.^4^^,^^5^^,^^6^^,^^7^^,^^8^ The aforementioned differences suggest that there may be differences in the molecular mechanisms regulating EMT in the two types of CRS. Existing research evidence suggests that EMT in CRSsNP is mainly driven by the TGF-β/Smad signaling pathway, while CRSwNP involves complex interactions of multiple pathways such as TGF-β, Wnt/β-catenin, hypoxia signaling, and MAPK/ERK.^9^^,^^10^^,^^11^^,^^12^^,^^13^^,^^14^^,^^15^^,^^16^^,^^17^ Therefore, elucidating key signaling mechanisms of EMT in CRS is of great clinical importance.

DNAJB9 is a multifunctional molecular chaperone protein involved in various biological processes, such as cellular stress response and metabolic regulation, and it has been confirmed to be a key molecule in cell differentiation and EMT regulation.^18^^,^^19^^,^^20^ For example, DNAJB9 has been shown to suppress the EMT phenotype in triple-negative breast cancer.^21^^,^^22^ DNAJB9 is expressed in a variety of human tissues, especially in cells with active secretory function; in the respiratory tract, it is mainly distributed in the nasal epithelium, bronchial epithelium, alveolar macrophages, and type II alveolar cells.^23^ However, the exact mechanism of DNAJB9 action in epithelial remodeling in CRS is not yet clear.

In this study, we confirmed that DNAJB9 is a critical regulator of EMT in nasal epithelium. *In vitro* and *in vivo* results have shown that DNAJB9 positively regulates the NF-κB signaling pathway: it promotes IκBα degradation through TRIM22-mediated ubiquitination, which, in turn, activates NF-κB signaling and promotes EMT progression. The aforementioned results suggest that targeted regulation of the DNAJB9-NF-κB signaling axis is expected to provide a potential therapeutic strategy for epithelial remodeling in sinusitis.

## Results

### DNAJB9 is highly expressed in CRS nasal epithelium

**Figure 1:**
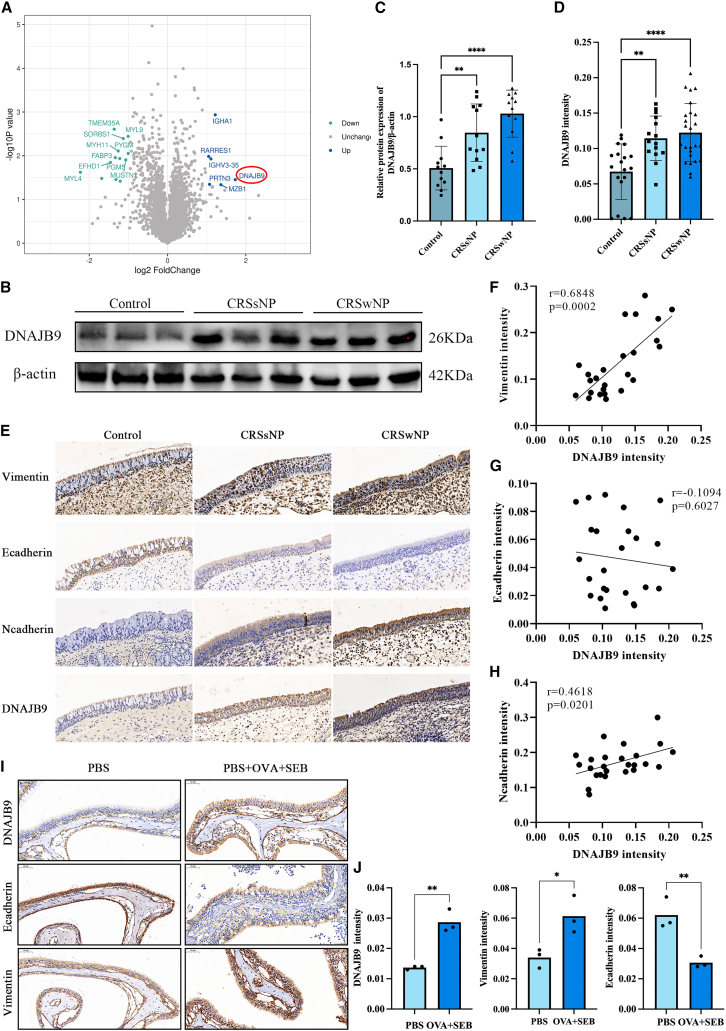
DNAJB9 is highly expressed in CRSwNP (A) Volcano plots of Differentially Expressed Proteins (DEPs) in CRSwNP group vs. HC group. Blue points represented upregulated proteins and green points represented downregulated proteins filtered based on fold change (FC) ≥2.0 and *p* < 0.05. (B) Western blot analysis was used to detect the protein expression levels of DNAJB9. β-Actin was used as a control. (C) Quantitative analysis of DNAJB9 protein levels. (D) Representative IHC staining of the expression and localization of vimentin, E-cadherin, N-cadherin, and DNAJB9 from the control, CRSsNP, and CRSwNP groups (magnification: 400×; scale bars, 50 μm). (E) Quantitative analysis of DNAJB9 staining intensity in nasal mucosa epithelium of each group. (F) Correlation analysis between DNAJB9 and vimentin expression intensity; r = 0.6848, *p* = 0.0002. (G) Correlation analysis between DNAJB9 and E-cadherin expression intensity; r = −0.1094, *p* = 0.6027. (H) Correlation analysis between DNAJB9 and N-cadherin expression intensity; r = 0.4618, *p* = 0.0201. (I) Representative IHC staining of the expression and localization of DNAJB9, E-cadherin, and vimentin from the control and CRSwNP mouse turbinate mucosa (magnification: 400×; scale bars, 50 μm). (J) Quantitative analysis of DNAJB9, E-cadherin, and vimentin staining intensity in turbinate mucosa epithelium of two groups. For all plots presenting error bars, data are presented as mean ± standard deviation (SD). Pearson correlation analysis was used for (F)–(H). Statistical significance was determined using unpaired Student’s *t* test for (J), and one-way ANOVA followed by Tukey’s post hoc test for (C) and (D). ∗*p* < 0.05, ∗∗*p* < 0.01, ∗∗∗*p* < 0.001.

To investigate the molecular changes of CRSwNP, proteomic differences were analyzed in nasal mucosa tissue samples from healthy controls and CRSwNP patients by using proteomics techniques in this study. There were no significant differences in clinical baseline data such as age, gender, disease duration, and comorbidities between the two groups (*P* > 0.05), as detailed in Table 1. Volcano plot analysis showed that DNAJB9 protein expression levels were significantly upregulated in the CRSwNP group compared with healthy controls (Figure 1A; see also Figures S2A and S2B, related to Figure 1). To verify the reliability of the aforementioned proteomic findings, we detected the expression levels of DNAJB9 protein in clinical nasal mucosa tissues by western blot; the results showed that DNAJB9 protein expression was significantly increased in CRSsNP and CRSwNP tissues compared with the normal control group, and DNAJB9 expression levels were highest in the CRSwNP group (Figures 1B and 1C). In agreement, quantitative immunohistochemistry (IHC) analysis further confirmed that DNAJB9 protein expression intensity was progressively upregulated from normal controls to CRSsNP tissues and then to CRSwNP tissues, and it was mainly localized in the epithelial layer of the nasal mucosa (Figures 1D and 1E).

**Table 1:** Clinical data

Variable	HC *N* = 22	CRSsNP *N* = 15	CRSwNP *N* = 25
Age (year), mean ± SD	47.0 ± 18.6	44.3 ± 14.8	47.6 ± 20.7
Male,N(%)	12(54.5%)	9(56.3%)	16(64.0%)
History of Prior Nasal Surgery,N(%)	3 (13.6%)	1 (6.3%)	4 (16.0%)
Asthma, N (%)	0 (0%)	0 (0%)	0 (0%)
Allergy,N(%)	0 (0%)	0 (0%)	0 (0%)
Smoking,N(%)	5 (22.7%)	3 (18.8%)	9 (36.0%)
Alcohol,N(%)	3 (13.6%)	1 (6.3%)	4 (16.0%)
Lund-Kennedy score	2.8 ± 1.8	5.3 ± 1.5	8.0 ± 2.2
peripheral blood eosinophils count (×10^9^/L)	0.23 ± 0.19	0.17 ± 0.17	0.21 ± 0.13
peripheral blood eosinophils percentage (%)	3.9 ± 3.0	2.8 ± 2.3	3.4 ± 2.4

HC, health control. CRSsNP, Chronic rhinosinusitis without nasal polyps. CRSwNP, Chronic rhinosinusitis with nasal polyps.

The IHC staining results showed that vimentin and N-cadherin, markers in the intermediate substance of CRSwNP tissues, showed a significant accumulation state accompanied by abnormal morphological changes in nasal mucosal epithelial cells (Figure 1E). To identify the correlation between DNAJB9 and EMT markers, we further carried out correlation analysis, and the results showed that the intensity of DNAJB9 expression was strongly positively correlated with vimentin, and the difference was statistically significant (r = 0.6848, *p* = 0.0002; Figure 1F); it was moderately positively correlated with N-cadherin, and the difference was also statistically significant (r = 0.4618, *p* = 0.0201; Figure 1H). However, although weak negative correlations were observed between DNAJB9 expression and epithelial marker E-cadherin in all clinical specimens, no significant statistical correlation was observed (r = −0.1094, *p* = 0.6027; Figure 1G).

To verify whether the aforementioned results observed in human tissues could be reproduced in *in vivo* experiments, we used OVA combined with SEB induction to establish a mouse model of CRSwNP and performed related tests on their nasal mucosal tissues (Figure S2C, related to Figure 1). The IHC staining results of polypoid nasal mucosa tissues of mice confirmed that vimentin expression was significantly increased, epithelial marker E-cadherin expression was significantly downregulated, and DNAJB9 protein expression intensity was significantly upregulated in the nasal mucosa of OVA + SEB-treated mice compared with PBS-treated controls, and the aforementioned results were consistent with the detection results in human tissues (Figures 1I and 1J). Collectively, these data indicate that aberrant DNAJB9 expression is a hallmark of CRSwNP and is closely associated with the EMT phenotype.

### DNAJB9 silencing inhibits EMT in nasal epithelial cells

**Figure 2:**
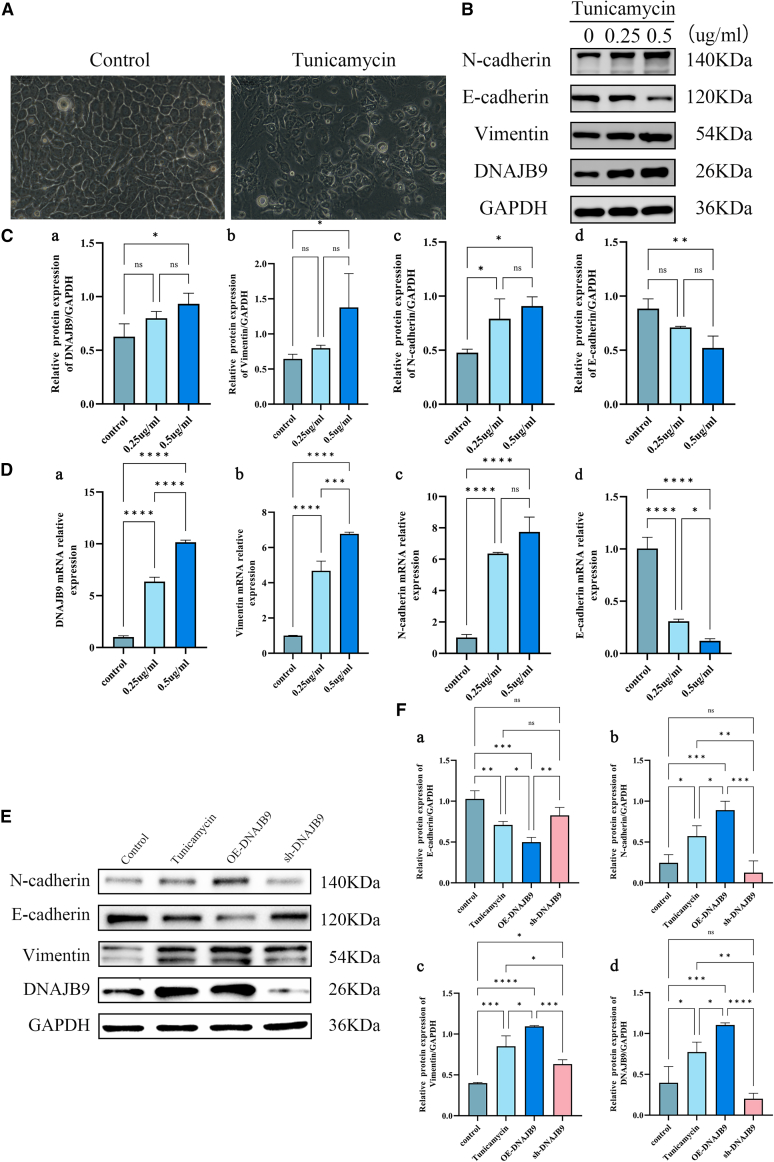
DNAJB9 promotes epithelial-mesenchymal transition in nasal epithelial cells (A) Comparison of primary nasal epithelial cell morphology between control and 0.5 μg/mL tunicamycin treated for 48 h (magnification: 400×; scale bars, 1000 μm). Tunicamycin treatment induced the transition of cells from epithelial-like to mesenchymal-like morphology. (B) hNECs were treated with tunicamycin at concentrations ranging from 0 to 0.5 μg/mL for 48 h. Western blot analysis was used to detect the protein expression levels of DNAJB9 and EMT-related markers. GAPDH was used as a control. (C) Quantitative analysis of DNAJB9 and EMT-related markers protein levels. (D) Quantitative analysis of the relative mRNA expression of DNAJB9 and EMT-related markers. (E) BEAS-2B cells were treated for 48 h under the conditions indicated in the figure, and western blot analysis was used to detect the protein expression levels of DNAJB9 and EMT-related markers. GAPDH was used as a control. (F) Quantitative analysis of DNAJB9 and EMT-related markers protein levels. Data in (C), (D), and (F) are presented as mean ± standard deviation (SD) from three independent experiments. Statistical significance was determined using one-way ANOVA followed by Tukey’s post hoc test. ∗*p* < 0.05, ∗∗*p* < 0.01, ∗∗∗*p* < 0.001.

Subsequently, we treated human nasal epithelial cells (hNECs) with tunicamycin and observed their morphological changes and changes in the expression of EMT-related markers. Microscopically, hNECs treated with tunicamycin showed a significant change in morphology, from regular paving stone-like epithelioid morphology to irregular morphology, compared with the control group (Figure 2A). Western blot and quantitative RT-qPCR showed that the protein and mRNA expression levels of DNAJB9 were significantly upregulated with the increase of tunicamycin stimulation concentration, accompanied by a significant increase in the protein and mRNA expression levels of mesenchymal markers N-cadherin and vimentin; correspondingly, the protein and mRNA expression levels of epithelial marker E-cadherin were significantly decreased (Figures 2B and 2D).

Given the correlation between elevated DNAJB9 expression and EMT occurrence observed in hNECs, we sought to further explore the specific function of DNAJB9 in this process. Based on the ‘one airway, one disease’ concept, we selected BEAS-2B cells as our research model. Compared with human primary nasal epithelial cells, BEAS-2B cells exhibit higher gene transduction efficiency and growth stability. This technical advantage ensures consistent transfection efficiency and high reproducibility for the OE-DNAJB9 and sh-DNAJB9 experiments used to verify the role of DNAJB9 in EMT (Figures S2D–S2F, related to Figure 2). The results showed that the levels of DNAJB9 protein in the cells were significantly increased after DNAJB9 overexpression, and the protein expression levels of N-cadherin and vimentin were further increased compared with tunicamycin-stimulated group and normal control group, while the expression levels of E-cadherin were further decreased, suggesting that DNAJB9 can induce EMT effect in nasal epithelial cells. Compared with tunicamycin-stimulated group and normal control group, DNAJB9 protein expression in cells was significantly inhibited after DNAJB9 knockdown, while the protein expression levels of N-cadherin and vimentin were significantly decreased, while the expression levels of E-cadherin were partially restored (Figures 2E and 2F). The aforementioned results demonstrate that DNAJB9 is a key molecule that induces EMT in nasal epithelial cells.

### DNAJB9 promotes EMT in nasal epithelium by activating the NF-κB signaling pathway

**Figure 3:**
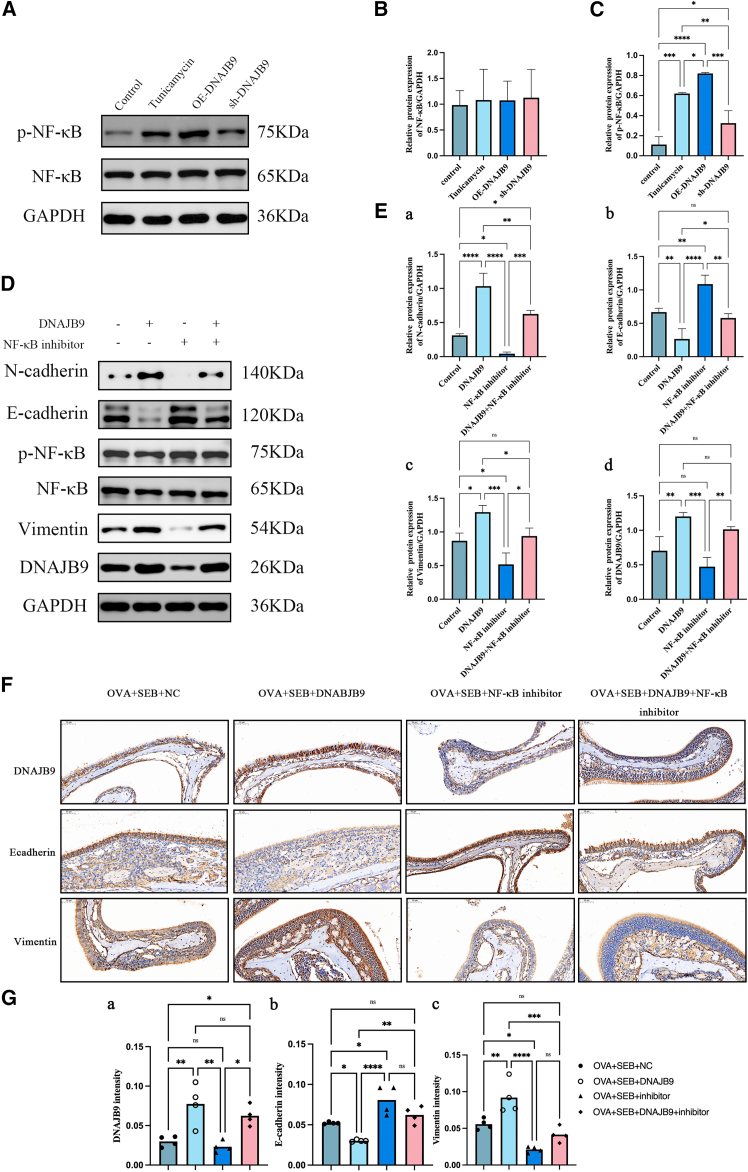
DNAJB9 drives EMT in nasal epithelial cells through activation of NF-κB signaling pathway (A) NF-κB p65 and its phosphorylation levels were detected by western blotting in BEAS-2B cells stably overexpressing or knocking down DNAJB9 for the indicated times. (B and C) Quantitative analysis of NF-κB and p-NF-κB protein levels. (D) Expression of DNAJB9, NF-κB, p-NF-κB, and EMT-related markers was detected by western blotting in BEAS-2B cells stably overexpressing DNAJB9 treated for 48 h with NF-κB pathway inhibitors. (E) Quantitative analysis of DNAJB9 and EMT-related markers protein levels. A mouse DNAJB9 overexpression model was constructed by intranasal instillation of AAV and intervened with or without NF-κB inhibitors. Specific groupings are shown. (F) Representative immunohistochemical staining showed the expression and localization of DNAJB9, epithelial marker E-cadherin, and mesenchymal marker vimentin in the turbinate mucosa of mice in each group (magnification: 400×; scale bars, 50 μm). (G) Quantitative analysis of DNAJB9, Ecadherin and vimentin staining intensity. Data in (B), (C), (E), and (G) are presented as mean ± standard deviation (SD). Statistical significance was determined using one-way ANOVA followed by Tukey’s post hoc test. ∗*p* < 0.05, ∗∗*p* < 0.01, ∗∗∗*p* < 0.001.

To explore the molecular mechanisms downstream of DNAJB9, we performed Kyoto Encyclopedia of Genes and Genomes (KEGG) pathway enrichment analysis, which highlighted the NF-κB signaling pathway as a potential target (Figure S3A, related to Figure 3). To clarify the regulation of NF-κB signaling pathway by DNAJB9, the mRNA expression level of RELA was first detected in this study, and the results showed that the mRNA expression level of RELA was significantly increased in cells overexpressing DNAJB9 (Figure S3B, related to Figure 3). Next, western blot showed that tunicamycin induction significantly increased the protein expression level of p-NF-κB compared with the control group; overexpression of DNAJB9 further upregulated the expression level of p-NF-κB, while knockdown of DNAJB9 significantly inhibited the phosphorylation process of NF-κB. Notably, there was no significant difference in total NF-κB protein expression levels between the groups (Figures 3A–3C). To verify the necessity of NF-κB pathway in DNAJB9-mediated EMT, reversion experiments were carried out using NF-κB inhibitors in this study. The results showed that overexpression of DNAJB9 alone significantly induced the upregulation of mesenchymal markers N-cadherin, vimentin, and p-NF-κB and significantly downregulated the expression of epithelial marker E-cadherin; after combined use of NF-κB inhibitors, DNAJB9-induced upregulation of N-cadherin and vimentin expression was significantly weakened, and the expression level of E-cadherin was partially restored (Figures 3D and 3E).

Pathological changes in nasal mucosal tissues were further observed by IHC staining after administration of different interventions in the CRSwNP mouse model. The results showed that after overexpression of DNAJB9 in the nasal mucosa of CRSwNP mice, the immunostaining intensity of vimentin was significantly enhanced compared with the control group, while the staining intensity of E-cadherin was further attenuated; after application of NF-κB inhibitors, the positive expression of vimentin in the turbinate mucosa was significantly reduced, and the expression level of E-cadherin was significantly restored (Figures 3F, 3G, and S3C, related to Figure 3). These findings establish that DNAJB9 drives nasal epithelial EMT primarily by activating the NF-κB signaling cascade.

### Ubiquitination of DNAJB9 degrades IKBa

**Figure 4:**
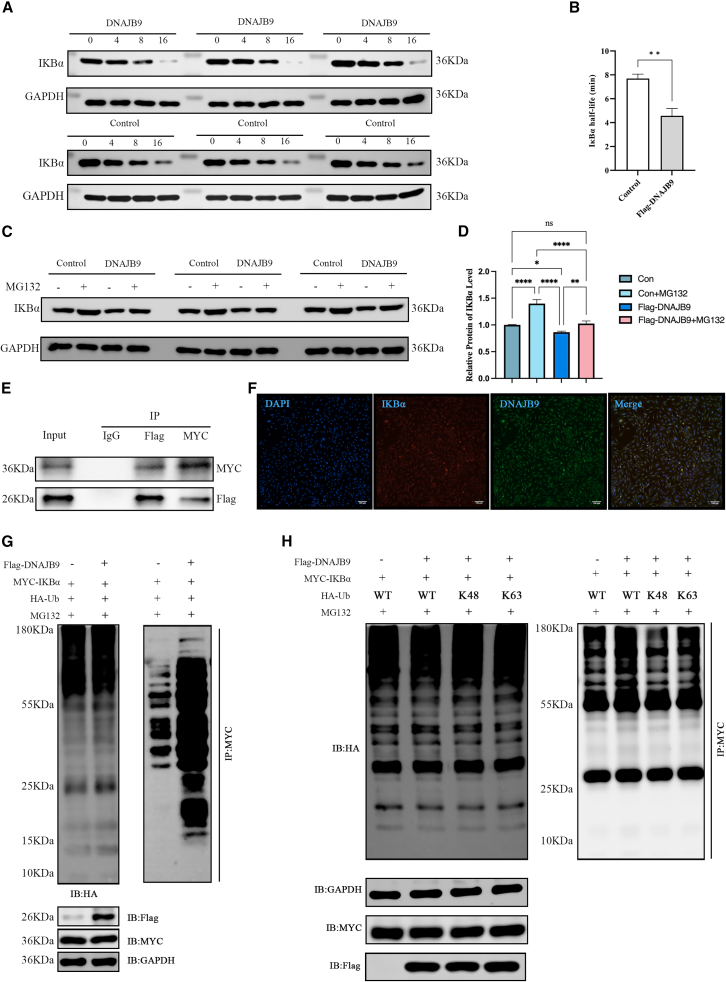
DNAJB9 promotes IκBα degradation in BEAS-2B cells (A) IκBα protein levels were detected by western blot and normalized to an internal reference GAPDH. Data are shown as mean ± standard deviation from three independent experiments. (B) Protein band intensities were quantified and normalized to GAPDH loading controls. The relative protein abundance at each time point (0, 4, 8, 16 min) was expressed relative to the 0 min time point (set as 1.0 or 100%). Degradation kinetics were analyzed using a one-phase exponential decay model defined by the equation: Y(t) = Y0·e^-kt^, t_1/2_ = (ln 2)/k. Half-life values were determined individually for each biological replicate (*n* = 3). Statistical significance between the control and Flag-DNAJB9 groups was analyzed using an unpaired two-tailed Student’s *t* test. (C) Control or DNAJB9-overexpressing BEAS-2B cells were treated with cycloheximide (CHX, 10 μm), and protein samples were collected at the 8 h time point with or without MG132 treatment. IκBα protein levels were detected by western blot and normalized to an internal reference GAPDH. (D) Quantitative analysis of IκBα protein levels. (E) Expression plasmids for Flag-DNAJB9 with Myc-IKBα were co-transfected in HEK293 cells, and cell lysates were collected for co-immunoprecipitation followed by detection of immune complexes by western blot. (F) After BEAS-2B cells were treated with 10 ng/mL TGF-β1 for 48 h, co-localization of IκBα (red) with DNAJB9 (green) was observed by immunofluorescence staining. Nuclei were stained with DAPI (blue). (G) HEK293 cells were transiently co-transfected with the control vector, Flag-DNAJB9, and with Myc-IKBα or HA-ubiquitin, as indicated. At 48 h post-transfection, the cells were incubated with MG132 for 6 h and then subjected to ubiquitination assay. In all, 10% of cell lysates were used for the analysis of input. (H) To determine the DNAJB9-mediated ubiquitination type of IKBα, DNAJB9 and IKBα constructs were transfected into HEK293 cells with various ubiquitin plasmids. Ubiquitinated IKBα was detected with anti-HA antibody from immunoprecipitated IKBα protein. For all quantitative panels (B and D), data are shown as mean ± standard deviation (SD) from three independent experiments. Statistical significance was analyzed using the unpaired two-tailed Student’s *t* test for B, and one-way ANOVA followed by Tukey’s post-hoc test for D. ∗*p* < 0.05, ∗∗*p* < 0.01, ∗∗∗*p* < 0.001.

Given that IκBα functions as the primary inhibitor of NF-κB by sequestering it in the cytoplasm, we investigated whether DNAJB9 regulates IκBα stability. DNAJB9 overexpression accelerated IκBα protein degradation without altering its mRNA abundance, pointing to a post-transcriptional regulatory mechanism (Figures 4A, 4B, and S3D, related to Figure 4). Indeed, treatment with the proteasome inhibitor MG132 effectively prevented DNAJB9-mediated IκBα reduction (Figure 4C).

Co-immunoprecipitation (co-IP) assays confirmed a direct physical interaction between DNAJB9 and IκBα (Figure 4E), which was further supported by their co-localization in immunofluorescence staining (Figure 4F). Mechanistically, ubiquitination assays demonstrated that DNAJB9 overexpression robustly enhanced the polyubiquitination of IκBα. Notably, this modification was predominantly K48-linked (Figures 4G and 4H), a canonical signal for proteasomal degradation. These results suggest that DNAJB9 activates NF-κB by facilitating the ubiquitin-proteasome degradation of its inhibitor, IκBα.

### DNAJB9 degrades IKBa through TRIM22

**Figure 5:**
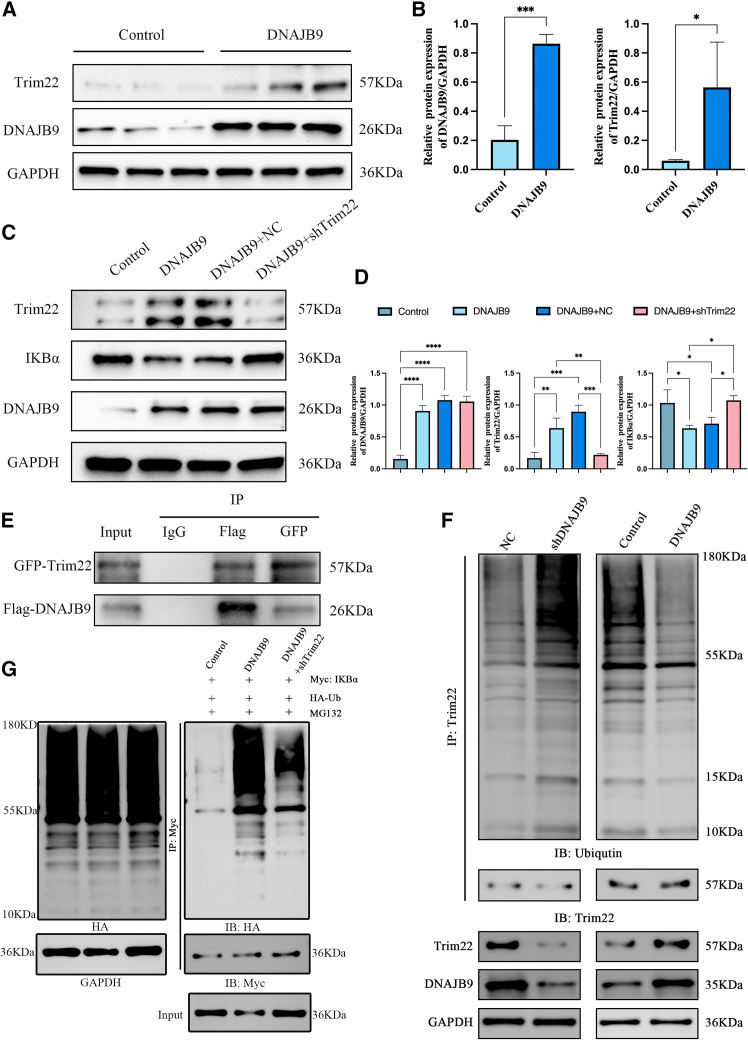
DNAJB9 promotes IκBα degradation through Trim22 dependent ubiquitination (A) Stably overexpressed BEAS-2B cells were stimulated with TGF-β1 for 48 h, and Western blot results showed TRIM22 protein expression levels. (B) Quantitative analysis of DNAJB9 and Trim22 protein levels. (C) Simultaneous knockdown of TRIM22 in DNAJB9-overexpressing BEAS-2B cells, Western blot results showed TRIM22 protein expression levels. (D) Quantitative analysis of DNAJB9, Trim22 and IκBα protein levels. (E) Expression plasmids for Flag-DNAJB9 with GFP-Trim22 were co-transfected in HEK293 cells, and cell lysates were collected for co-immunoprecipitation followed by detection of immune complexes by Western blot. (F) To verify the inhibitory effect of DNAJB9 on TRIM22 ubiquitination, we immunoprecipitated lysates from BEAS-2B cells stably silenced or overexpressing DNAJB9 using anti-TRIM22 antibody, respectively, and subsequently detected the ubiquitination level of TRIM22 obtained from the pellet using anti-ubiquitin antibody.G To verify whether TRIM22 mediates IκBα ubiquitination, we co-transfected IκBα with ubiquitin expression plasmids in HEK293 cells under other conditions as shown, followed by co-immunoprecipitation of IκBα using anti-HA antibody to analyze its ubiquitination level. For all quantitative panels (B and D), data are shown as mean ± standard deviation (SD) from three independent experiments. Statistical significance was analyzed using the unpaired two-tailed Student’s *t* test for B, and one-way ANOVA followed by Tukey’s post-hoc test for D. ∗*p* < 0.05, ∗∗*p* < 0.01, ∗∗∗*p* < 0.001.

Building on the concept that molecular chaperones often cooperate with E3 ubiquitin ligases to target substrates,.^24^^,^^25^^,^^26^ we hypothesized that DNAJB9 might engage the E3 ligase TRIM22 to degrade IκBα. We found that DNAJB9 overexpression increased both the mRNA and protein levels of TRIM22 (Figures 5A, 5B, and S3E, related to Figure 5). Crucially, knockdown of TRIM22 rescued the IκBα levels that were suppressed by DNAJB9 overexpression (Figures 5C, 5D, and S3F–S3H, related to Figure 5), indicating that TRIM22 is the downstream effector.

Co-IP experiments revealed a direct interaction between DNAJB9 and TRIM22 (Figure 5E). Further mechanistic analysis unveiled that DNAJB9 binding significantly inhibited the autoubiquitination of TRIM22 (Figure 5F), thereby enhancing its stability. Finally, ubiquitination assays showed that while DNAJB9 promotes IκBα ubiquitination, this effect was markedly blunted upon TRIM22 knockdown (Figure 5G). Collectively, these data delineate a molecular axis: DNAJB9 binds and stabilizes TRIM22 by preventing its autoubiquitination; stabilized TRIM22 then targets IκBα for K48-linked polyubiquitination and degradation, leading to constitutive NF-κB activation.

## Discussion

EMT is a key pathological process in which epithelial cells lose their apical and basal polarity and intercellular junctions and obtain cell migration ability. In CRS, especially CRSwNP, EMT is closely related to epithelial barrier dysfunction, chronic inflammatory stimuli, and tissue fibrosis.^5^^,^^8^^,^^27^^,^^28^^,^^29^ Further analysis of the regulatory molecular network of EMT in CRS not only helps to elucidate the heterogeneous characteristics of the disease but also provides important theoretical support for exploring therapeutic intervention targets. In this study, we systematically investigated the central role of DNAJB9 in the pathogenesis of CRSwNP in combination with clinical histological evidence and *in vitro* and *in vivo* functional experiments. The main findings showed that DNAJB9 was highly expressed in the nasal mucosa of patients with CRSwNP and in mouse nasal polyp models, and its expression level was significantly positively correlated with the interstitial marker vimentin; at the mechanistic level, DNAJB9 activated the NF-κB signaling pathway by promoting IκBα degradation and then induced EMT in nasal epithelial cells. Notably, this regulatory process is highly dependent on the E3 ubiquitin ligase TRIM22, which promotes its degradation via the proteasome pathway mainly by mediating K48-linked polyubiquitination modification of IκBα. Based on the above findings, a molecular regulatory axis, DNAJB9-TRIM22-IκBα-NF-κB, is proposed for the first time in this study, which provides a brand-new theoretical perspective for elucidating the pathogenesis of CRSwNP.

In order to investigate the pathogenic mechanism of CRSwNP more deeply, in this study, we first used Tandem Mass Tags (TMT) quantitative proteomics to differentially analyze nasal mucosa tissues and identified DNAJB9 as abnormally enriched in CRSwNP tissues. Combined with clinical sample detection, animal model validation, and cytological experiments, this study clearly confirms for the first time the key role of DNAJB9 in driving the EMT process of nasal epithelium. In addition to the aforementioned expression correlation, *in vitro* experiments further confirmed that tunicamycin stimulation significantly induced the upregulation of DNAJB9 expression in primary nasal epithelial cells; gain-of-function and deletion experiments showed that overexpression of DNAJB9 significantly promoted EMT phenotype formation, while knockdown of DNAJB9 effectively blocked this process. Interestingly, the conclusions of this study are diametrically opposite to previous literature reports of DNAJB9 function in inhibiting EMT in triple-negative breast cancer, suggesting that the biological effects of DNAJB9 are highly tissue-specific and microenvironment-dependent.^21^ We speculate that DNAJB9 may tend to drive EMT development by stabilizing specific transcription factors in the chronic inflammatory microenvironment of CRSwNP; in the malignant tumor microenvironment, its core function is to maintain cellular proteostasis, thereby inhibiting abnormal cell proliferation. This phenomenon not only highlights the complexity of molecular chaperone protein function but also suggests that DNAJB9 has heterogeneous therapeutic targeting potential in different disease contexts.

NF-κB, as a core transcription factor in the body, can induce EMT by promoting the release of inflammatory factors in a variety of respiratory diseases, which, in turn, exacerbates airway remodeling and tissue fibrosis.^30^^,^^31^^,^^32^^,^^33^ Inhibition of this pathway has been shown to be effective in delaying EMT and fibrotic progression.^34^^,^^35^ Based on the results of KEGG pathway enrichment analysis of omics data, combined with the critical role of NF-κB in innate immune responses, this study focused on NF-κB signaling pathways. The results of *in vitro* and *in vivo* experiments consistently showed that blocking NF-κB activity significantly alleviated the deteriorating process of EMT in the nasal epithelium. Mechanistic studies showed that overexpression of DNAJB9 significantly upregulated p-NF-κB expression levels, while knockdown of DNAJB9 inhibited NF-κB activation; more importantly, introduction of NF-κB-specific inhibitors effectively reversed the EMT phenotype induced by DNAJB9 overexpression. The aforementioned results fully confirmed that activation of NF-κB signaling pathway is necessary for DNAJB9 to drive EMT in nasal epithelium, further validating the scientific hypothesis that “DNAJB9-NF-κB signaling cascade promotes EMT in nasal epithelium”.

As a classical molecular chaperone, DNAJB9 specifically recognizes client proteins and recruits them to the ubiquitin-proteasome system to mediate degradation.^36^^,^^37^ Given that IκBα is a core negative regulator of the NF-κB signaling pathway, this study further investigated the interaction relationship between DNAJB9 and IκBα in depth.^38^^,^^39^^,^^40^^,^^41^ The results showed that DNAJB9 overexpression significantly shortened the protein half-life of IκBα, and co-IP experiments clearly confirmed that there was a direct binding between the two in cells. Critically, DNAJB9 does not affect IκBα transcript levels but directly promotes K48-linked polyubiquitination modification of IκBα, thereby accelerating its degradation via the proteasome pathway. These findings clarify the core role of DNAJB9 as an upstream regulatory molecule of the NF-κB pathway and provide a brand-new experimental basis for its specific molecular mechanism of activating the NF-κB pathway.

Further investigation revealed that DNAJB9-mediated polyubiquitination of IκBα (K48-linked) was significantly increased, suggesting the involvement of specific E3 ubiquitin ligases. TRIM22 has been documented as an E3 ubiquitin ligase that catalyzes K48-linked ubiquitination modification of IκBα and accelerates its degradation.^26^^,^^42^^,^^43^^,^^44^^,^^45^ In this study, we confirmed that overexpression of DNAJB9 significantly upregulated the protein expression level of TRIM22, while knockdown of TRIM22 reversed IκBα degradation induced by DNAJB9; co-IP experiments further confirmed that there was a direct interaction between DNAJB9 and TRIM22. Deep mechanistic dissection suggests that DNAJB9 inhibits its autoubiquitination process by binding TRIM22, thereby maintaining the stability of TRIM22 protein, which, in turn, amplifies its ubiquitination efficacy against IκBα. Ubiquitination experiments also confirmed that knockdown of TRIM22 significantly attenuated the promoting effect of DNAJB9 overexpression on IκBα ubiquitination. In summary, DNAJB9 synergistically enhances K48 polyubiquitination modification of IκBα by TRIM22 and subsequent degradation processes by directly binding to and inhibiting autoubiquitination degradation of TRIM22.

This study has important scientific significance and potential clinical translational value for the development of CRSwNP pathogenesis and targeted therapy fields. First, in this study, DNAJB9 was clearly identified as a key driver of tissue remodeling in CRSwNP by TMT quantitative proteomics technology for the first time, which significantly expanded the cognitive boundary of the academic community on molecular chaperone protein function in chronic inflammation of the upper respiratory tract, and provided a characterized entry point for subsequent studies in this field. Secondly, this study system reveals a fine molecular regulatory pathway, the “DNAJB9-TRIM22-IκBα axis,” and successfully establishes a tight association bridge between chaperone-mediated proteostasis and activation of the canonical NF-κB inflammatory pathway; particularly important, DNAJB9 can play a regulatory role by antagonizing TRIM22 autoubiquitination, a finding that provides a previously unreported perspective for elucidating the post-translational modification regulatory mechanism of E3 ubiquitin ligases during EMT in CRSwNP and enriches the theoretical study of EMT regulatory networks. Finally, this study clearly confirms that the targeted DNAJB9-TRIM22 interaction interface has great application potential in intervening the pathological process of nasal polyps, providing a promising alternative strategy for accurate targeted therapy of CRSwNP, and also laying a solid theoretical foundation for subsequent related drug research and development.

### Limitations of the study

Although a series of meaningful findings have been made in this study, there are still limitations that need to be further refined in subsequent studies. First, BEAS-2B cell line was mainly used for *in vitro* mechanism validation. Although this cell line could better reflect the common biological characteristics of airway epithelium, it was still different from primary nasal epithelial cells. In future, it is necessary to further verify the relevant mechanisms in primary nasal epithelial cells to more accurately simulate the local inflammatory microenvironment of the nasal mucosa and improve the tissue specificity and clinical relevance of the findings. Second, limited by the small size of the currently included clinical samples, this study failed to fully analyze the direct correlation between DNAJB9 expression levels and patient-reported outcomes measures (such as the Sinonasal Outcome Test-22 scale (SNOT-22), visual analogue scale (VAS), and typical clinical symptoms such as anosmia and nasal obstruction). Given that EMT is closely related to the refractory and high recurrence rate of CRSwNP, subsequent studies will expand the sample size and include long-term follow-up data to deeply investigate whether high DNAJB9 expression can be used as a potential biomarker to predict the risk of postoperative recurrence in patients, as well as resistance to standard therapeutic drugs such as corticosteroids. Third, the exact domain and binding interface of DNAJB9 and TRIM22 interaction have not been fully elucidated, and the fine mechanism of their molecular interaction still needs to be further explored and analyzed by structural biology techniques to provide a more accurate theoretical basis for drug research and development targeting the interaction interface between the two.

## Resource availability

### Lead contact

Further information and requests for resources and reagents should be directed to and will be fulfilled by the lead contact, Chunping Yang (ndefy09036@ncu.edu.cn).

### Materials availability

This study did not generate new unique reagents.

### Data and code availability

• The mass spectrometry proteomics data generated during this study have been deposited to the ProteomeXchange Consortium via the PRIDE partner repository and are publicly accessible. The dataset identifier is PXD079333.
• This paper does not report original code.
• Any additional information required to reanalyze the data reported in this paper is available from the lead contact upon request.

## Data availability

Data are available via ProteomeXchange with identifier PXD079333.

## Acknowledgments

The authors would like to express their sincere gratitude to Dr. Cheng Chiwen of The Second Affiliated Hospital of Nanchang University for his valuable experimental assistance. The authors also extend their thanks to the Experimental Platforms of Otolaryngology-Head and Neck Surgery and Anesthesiology at The First Affiliated Hospital of 10.13039/501100004637Nanchang University for their generous support. This study was supported by the 10.13039/501100001809National Natural Science Foundation of China (grant no. 82160211), the 10.13039/501100004479Jiangxi Provincial Natural Science Foundation (grant no. 20252BAC250088), and the National Natural Science Foundation Incubation Program of the Second Affiliated Hospital of 10.13039/501100004637Nanchang University (grant no. 2025YNFY12007).

## Author contributions

Z. Z and C. Y designed and performed the experiments. X. H and M. L collected clinical tissue samples and data. Z.Z, J. T, X. Z, and Y. W analyzed the data; Z. Z and X. H wrote the manuscript. C. Y supervised the project. All authors read and approved the final manuscript.

## Declaration of interests

The authors declare no competing interests.

## STAR★Methods

### Key resources table

**Table:** 

REAGENT or RESOURCE	SOURCE	IDENTIFIER
**Antibodies**		

DNAJB9	Proteintech	Cat# 13157-1-AP; RRID: AB_2877918
Vimentin	Huabio	Cat# ET1610-39; RRID: AB_3069923
E-cadherin	Proteintech	Cat# 20874-1-AP; RRID: AB_10697811
N-cadherin	Huabio	Cat# ER0503; RRID: AB_3069012
p65	Proteintech	Cat# 10745-1-AP; RRID: AB_2178878
pP65	Proteintech	Cat# 82335-1-RR; RRID: AB_3083091
Ikbα	Proteintech	Cat# 10268-1-AP; RRID: AB_2151423
Ikbα	CST	Cat# 4814; RRID: AB_390781
TRIM22	Proteintech	Cat# 13744-1-AP; RRID: AB_2877974
Flag	Proteintech	Cat# 20543-1-AP; RRID: AB_11232216
Flag	Proteintech	Cat# 66008-4-Ig; RRID: AB_2918475
MYC	Proteintech	Cat# 16286-1-AP; RRID: AB_11182162
MYC	Proteintech	Cat# 60003-2-Ig; RRID: AB_2734122
GFP	Proteintech	Cat# 50430-2-AP; RRID: AB_11042881
GFP	Proteintech	Cat# 66002-1-Ig; RRID: AB_11182611
HA	Proteintech	Cat# 51064-2-AP; RRID: AB_11042321
HA	Proteintech	Cat# 66006-2-Ig; RRID: AB_2881490
Ubiquitin	Proteintech	Cat# 10201-2-AP; RRID: AB_671515
GAPDH	Proteintech	Cat# 10494-1-AP; RRID: AB_2263076
β-Actin	Huabio	Cat# R1207-1; RRID: AB_3073201
IgG	Proteintech	Cat# SA00001-2; RRID: AB_2722564
IgG	Proteintech	Cat# SA00001-1-A; RRID: AB_2890995

**Bacterial and virus strains**		

Lentiviral particles expressing shRNA targeting DNAJB9 (pLKO.1)	Zaiji Biotechnology	NA
Lentiviral particles expressing shRNA targeting TRIM22 (pLKO.1)	Zaiji Biotechnology	NA
Lentiviral particles expressing targeting DNAJB9 (pCAG)	Zaiji Biotechnology	NA
Adenovirus expressing DNAJB9	Applied Biological Materials	NA

**Biological samples**		

Nasal mucosa	This paper	NA
Nasal polyp	This paper	NA
Mouse serum	This paper	NA

**Chemicals, peptides, and recombinant proteins**		

Tunicamycin	MCE	Cat# HY-A0098
MG132	Sigma-Aldrich	Cat# M8699
Cycloheximide	Sigma-Aldrich	Cat# 239763-M
infection enhancement solution	GeneChem	Cat# GeneChem
puromycin	MCE	Cat# HY-K1057
ovalbumin	Sigma-Aldrich	Cat# S7951
Staphylococcus aureus enterotoxin B	MCE	Cat# HY-P71808
NF-κB inhibitor	MCE	Cat# BAY 11-7082

**Critical commercial assays**		

BeyoMagTM Protein A + G	Beyotime	Cat# P2179M
BCA protein assay kit	Beyotime	Cat# P0010S
High efficiency transfection reagent	ZETA life	Cat# AD600025
IgE ELISA kit	Jianglai Biological	Cat# JL12885-48T

**Deposited data**		

Mass spectrometry proteomics dataset	This paper; PRIDE/ProteomeXchange Consortium	ProteomeXchange: PXD079333

**Experimental models: cell lines**		

Human Nasal Epithelial Cells	This paper	NA
HEK-293	Zhongqiao Xinzhou Biotechnology	NA
BEAS-2b	Zhongqiao Xinzhou Biotechnology	NA

**Experimental models: organisms/strains**		

BALB/c mice		NA

**Oligonucleotides**		

shDNAJB9-1 sequences (5′ to 3′) CTCAGATGCTAATAGACGAAA	Zaiji Biotechnology	NA
shDNAJB9-2 sequences (5′ to 3′) GCAGATTGACTCAAAGAAGAT	Zaiji Biotechnology	NA
shDNAJB9-3 sequences (5′ to 3′) GCTACAAGAAGCTCAACATCA	Zaiji Biotechnology	NA
shTrim22-1 sequences (5′ to 3′) CCAGATGCCGATTAGGTCGG	Zaiji Biotechnology	NA
shTrim22-2 sequences (5′ to 3′) CCGCATAAACGAGGTGGTCA	Zaiji Biotechnology	NA
shTrim22-3 sequences (5′ to 3′) GCACCTACGAGATCAAGATAA	Zaiji Biotechnology	NA
shNC sequences (5′ to 3′) UUCUCCGAACGUGUCACGUTT	Zaiji Biotechnology	NA
GAPDH Forward primer(5′ to 3′) GTCTCCTCTGACTTCAACAGCG	Zaiji Biotechnology	NA
GAPDH Reverse primer(5′ to 3′) ACCACCCTGTTGCTGTAGCCAA	Zaiji Biotechnology	NA
β-actin Forward primer(5′ to 3′) TGGCACCCAGCACAATGAA	Zaiji Biotechnology	NA
β-actin Reverse primer(5′ to 3′) CTAAGTCATAGTCCGCCTAGAAGCA	Zaiji Biotechnology	NA
E-cadherin Forward primer(5′ to 3′) GCCTCCTGAAAAGAGAGTGGAAG	Zaiji Biotechnology	NA
E-cadherin Reverse primer(5′ to 3′) TGGCAGTGTCTCTCCAAATCCG	Zaiji Biotechnology	NA
N-cadherin Forward primer(5′ to 3′) CCTCCAGAGTTTACTGCCATGAC	Zaiji Biotechnology	NA
N-cadherin Reverse primer(5′ to 3′) GTAGGATCTCCGCCACTGATTC	Zaiji Biotechnology	NA
Vimentin Forward primer(5′ to 3′) GAGGAAGCCGAAAACACCCT	Zaiji Biotechnology	NA
Vimentin Reverse primer(5′ to 3′) TTGCGTTCAAGGTCAAGACG	Zaiji Biotechnology	NA
DNAJB9 Forward primer(5′ to 3′) AGGACAAAGAGGTAGTGGAAGT	Zaiji Biotechnology	NA
DNAJB9 Reverse primer(5′ to 3′) CCTGGCGTGTCTGGAAATGA	Zaiji Biotechnology	NA
Trim22 Forward primer(5′ to 3′) GCTGTGCCTCCCTGTCGTATTG	Zaiji Biotechnology	NA
Trim22 Reverse primer(5′ to 3′) ATGAGTGCTCCGTGGTTTGTGAC	Zaiji Biotechnology	NA
NFKBIA Forward primer(5′ to 3′) CCCGCACCTCCACTCCATCC	Zaiji Biotechnology	NA
NFKBIA Reverse primer(5′ to 3′) AGCATTGACATCAGCACCCAAG	Zaiji Biotechnology	NA
RELA Forward primer(5′ to 3′) TGTGAAGAAGCGGGACCTGGAG	Zaiji Biotechnology	NA
RELA Reverse primer(5′ to 3′) AAGCAGAGCCGCACAGCATTC	Zaiji Biotechnology	NA

**Recombinant DNA**		

Flag-DNAJB9	Zaiji Biotechnology	NA
GFP-TRIM22	Zaiji Biotechnology	NA
MYC-IKBα	Zaiji Biotechnology	NA
HA-Ub	Zaiji Biotechnology	NA

**Software and algorithms**		

ImageJ	ImageJ	https://imagej.net/ij/
GraphPad Prism10	GraphPad	https://www.graphpad.com
slider view	slider view	https://www.3dhistech.com/software
Proteome Discoverer	Thermo Scientific	https://www.thermofisher.com/proteomediscoverer

**Other**		

BlasTaq™ 2X qPCR MasterMix	Applied Biological Materials	Cat# G891
5X All-In-One RT MasterMix	Applied Biological Materials	Cat# G490
Super ECL Plus	UElandy	Cat# S6009M
DAPI	SolarBio	Cat# S2110

### Experimental model and study participant details

#### Patients and tissue samples

All study participants were recruited from the Department of Otolaryngology-Head and Neck Surgery at The Second Affiliated Hospital of Nanchang University. The study was approved by the Ethics Committee of The Second Affiliated Hospital of Nanchang University, and all procedures were performed in strict compliance with the relevant regulatory standards and the Declaration of Helsinki **(Approval No. O-Yi Yan Lun Shen [2025] No. 122)**. Written informed consent was obtained from each participant before the commencement of any experiments.

A total of 22 healthy Controls (HC), 25 CRSwNP patients, and 15 CRSsNP patients were enrolled, with detailed clinical data presented in Table 1. The cohorts comprised both male and female adult subjects, with ages ranging from 26 to 68 years (mean ± SD: HC, 47.0 ± 18.6 years; CRSsNP, 44.3 ± 14.8 years; CRSwNP, 47.6 ± 20.7 years) as broken down in Table 1. The diagnosis of CRSwNP was made in accordance with the Chinese guidelines for the treatment of rhinosinusitis.^46^ The control subjects were individuals undergoing septoplasty for anatomical variations, with no other sinonasal diseases. During surgery, nasal polyp tissue was collected from CRSwNP patients, while middle turbinate mucosal tissue was obtained from control subjects. Given the balanced representation across our clinical sample groups, the potential influence or association of sex and gender on the expression profile of DNAJB9 and tissue remodeling was evaluated; however, no statistically significant sex- or gender-specific differences in the molecular clinical outcomes were observed in this study.

#### Experimental animals

All animal experimental protocols were approved by the Animal Ethics Committee of The First Affiliated Hospital of Nanchang University (Approval No: CDYFY-IACUC-202403QR006) and were strictly conducted in conformance with national and international regulatory standards for the care and use of laboratory animals. Male wild-type 6-week-old BALB/c mice (young adults, 20–25 g) were housed in sterile individually ventilated cages (IVCs) under a controlled 12-h light/dark cycle with *ad libitum* access to food and water.

#### Cell culture

Three cell types were used for experiments in this study. As previously described, hNECs were isolated from nasal mucosa tissues obtained from both male and female adult donors.^47^ Human bronchial epithelial cells (BEAS-2B cells, sex: male, derived from a non-cancerous autopsy donor) and human embryonic kidney cells (HEK 293 cells, sex: female) were purchased from Shanghai Zhongqiao Xinzhou Biotechnology Co., Ltd., China. Both commercial cell lines (BEAS-2B and HEK 293) were strictly authenticated by Short Tandem Repeat (STR) profiling by the supplier before use. Additionally, all cell lines were routinely tested for mycoplasma contamination using PCR-based assays and were confirmed to be completely free of mycoplasma contamination throughout the study. All cells were cultured at 37°C in a humidified incubator containing 5% CO_2_.

### Method details

#### Quantitative real-time PCR (RT-qPCR)

Total RNA was extracted … using TRIzol reagent (T9108, Takara). One μg of total RNA was reverse transcribed into cDNA using the All-in-One RT mix with DNase (R2020S, US EVERBRIGHT, Suzhou, China). The mRNA levels of target genes were quantified using SYBR Green and a CFX Connect system (Bio-Rad, CA, USA). Thermal cycling conditions were as follows: initial denaturation at 95°C for 3 min, followed by 40 cycles of 95°C for 10 s and 60°C for 30 s. Gene expression levels were calculated using the 2 ^(−ΔΔCt)^ method. Detailed information on primer sequences can be found in Table S1.

#### Western blot

For the Western blot assay, protein samples were separated by SDS-PAGE and transferred onto nitrocellulose membranes. The membranes were blocked with 5% non-fat milk in TBS-T buffer at room temperature for 1 h to prevent non-specific binding. Subsequently, the membranes were incubated with the appropriate primary antibodies diluted in 5% BSA in TBS-T (dilution ratios are detailed in Table S2) at 4°C overnight. After washing three times with TBS-T buffer, the membranes were incubated with corresponding HRP-conjugated secondary antibodies (dilution ratios are detailed in Table S1) at room temperature for 90 min. Target proteins were detected using an ECL chemiluminescence substrate kit and exposed using a ChemiDoc MP Imaging System (Bio-Rad, Hercules, CA, USA).

#### Cell transfection

In 293T cells, transient transfection of various plasmids was performed using Zeta Life transfection reagent (Zeta Life, USA) with a DNA-to-reagent ratio of 1:1 (μg/μL). The transfection medium was replaced with fresh complete medium 6 h post-transfection.

To generate stable DNAJB9-overexpressing and shRNA-knockdown cell lines, pre-packaged lentiviral particles were purchased from company. BEAS-2B cells were seeded in 6-well plates and infected with the lentiviral particles at an optimal multiplicity of infection (MOI = 10) in the presence of GeneChem infection enhancement solution. After 24 h of infection, the medium was replaced with fresh complete culture medium. For the construction of combined models (e.g., DNAJB9 overexpression followed by TRIM22 knockdown), cells first underwent stable DNAJB9-overexpression selection, followed by secondary infection with sh-TRIM22 lentiviral particles.

Forty-eight hours post-infection, cells were subjected to antibiotic selection using 2 μg//mL puromycin (Sigma-Aldrich. USA). The selection medium was refreshed every 48 h for 14 days until stable cell pools were established. The efficiency of stable overexpression and knockdown was verified by RT-qPCR and western blotting prior to all functional assays.

#### Histology and immunohistochemistry (IHC)

Human nasal mucosa tissues or mouse heads were fixed, embedded, and sectioned. The paraffin blocks were cut into 4 μm thick consecutive sections using a microtome, with the maximum cross-sectional area of each section. To evaluate the morphological characteristics of the tissues, hematoxylin and eosin (H&E) staining was performed (all reagents purchased from Seville Biotechnology Co., Ltd.). After conventional dewaxing and rehydration, the sections were stained with hematoxylin, followed by washing and eosin staining. Following staining, the sections were dehydrated and cleared with xylene. The samples were then mounted and scanned using a Pannoramic MIDI to analyze the morphological features of the tissue sections. To detect EMT markers in the tissues, IHC was employed. After dewaxing and rehydration, sections were treated with 3% hydrogen peroxide (H_2_O_2_) to block endogenous peroxidase activity. The sections were then blocked to prevent nonspecific binding and incubated with primary antibodies (Wuhan Boster, SA1023), with antibody concentrations listed in the Table S2. The sections were subsequently incubated with species-specific secondary antibodies (HRP-labeled), followed by color development using diaminobenzidine (DAB, Immunoway SW1030). After the color development reaction, sections were counterstained. All procedures were performed under optimized temperature and timing conditions. Immunostaining results were scanned using a Pannoramic MIDI system. Five random fields were recorded for each sample, and ImageJ software was used for further statistical analysis.

#### Cellular immunofluorescence

The cells were then fixed with fresh 4% paraformaldehyde for 15 min, permeabilized with 0.1% Triton X-100 for 10 min, and blocked with 1% bovine serum albumin for 30 min. DNAJB9 and IκBα antibodies were diluted to 1:200 and incubated with the cells overnight at 4°C. The following day, a mixed dilution of the two secondary antibodies was incubated with the cells in a dark, humidified environment for 2 h. The cells were then mounted on slides using anti-fade mounting medium containing DAPI (S2110, SolarBio) and observed and imaged using using a fluorescence microscope.

#### Total protein extraction

Cells and nasal mucosa tissues were lysed on ice for 30 min using a potent lysis buffer (R0020, SolarBio) supplemented with protein phosphatase inhibitors (P002, NCM Biotech). Total protein was extracted by centrifugation at 12,000 g for 10 min at 4°C after treatment with an ultrasonic homogenizer. Protein concentrations were measured using a BCA protein assay kit (P0010S, Beyotime).

#### Protein half-life analysis

To determine the half-life of IκBα, BEAS-2B cells with or without DNAJB9 overexpression were treated with 10 μM cycloheximide (CHX, Sigma-Aldrich) to inhibit *de novo* protein synthesis. Cells were harvested at 0, 2, 4, 8, and 16 h post-CHX treatment. Total protein was extracted using a potent lysis buffer supplemented with protein phosphatase inhibitors. Endogenous IκBα protein levels were detected via western blotting and normalized to GAPDH levels to correct for loading variations. The relative protein abundance at each time point was calculated by setting the expression level at 0 h to 100%. Degradation kinetics were analyzed using a one-phase exponential decay model defined by the equation: Y(t) = Y0·e-kt, t1/2 = (ln 2)/k. Half-life values were calculated independently for each biological replicate (*n* = 3).

#### Ubiquitination assay

For *in vitro* ubiquitination analysis, HEK293T cells were transfected as described above. At 48 h post-transfection, cells were treated with 10 μM MG132 (Sigma-Aldrich) for 6 h to inhibit proteasomal degradation. Cells were harvested and lysed using Western/IP Cell Lysis Buffer (R0100, Solarbio) supplemented with protease and phosphatase inhibitors. The lysates were centrifuged at 12,000 × g for 15 min at 4°C, and the supernatants were collected. For immunoprecipitation, lysates were incubated with an anti-TRIM22 antibody (dilution ratios are detailed in Table S2) at 4°C for 24 h with gentle rotation. Subsequently, Protein A/G magnetic beads (Beyotime) were added, and the mixture was incubated for an additional 2 h at 4°C. The bead-antigen-antibody complexes were washed three times with ice-cold PBS to remove non-specific binding. Finally, the immunoprecipitated proteins were eluted by boiling in SDS-PAGE loading buffer, separated by SDS-PAGE, and analyzed by western blotting using an anti-ubiquitin antibody (dilution ratios are detailed in Table S2).

#### Animal experiments

A CRSwNP mouse model was established using ovalbumin (OVA; Sigma S7951) and Staphylococcus aureus enterotoxin B (SEB; MCE HY-P71808). Briefly, on days 0 and 5, mice received intraperitoneal injections of 25 μg OVA dissolved in 200 μL PBS containing 2 mg aluminum hydroxide (Sigma 239186). From day 12–19, mice received daily intranasal instillations of 40 μL of 6% OVA. Subsequently, from day 20–47, intranasal instillations of 40 μL of 6% OVA were administered three times per week. From day 48–103, mice received intranasal instillations of 40 μL of 6% OVA combined with 10 ng SEB three times per week. The treatment groups were assigned as follows: the control group received an equal volume of intranasal PBS; the experimental groups received OVA + SEB alongside therapeutic interventions. Interventions included PBS, an NF-κB inhibitor (MCE, BAY 11–7082; 5 mg/kg, three times per week from day 48–103), or lentiviral vectors (negative control or OE-DNAJB9; 1×10^11^vg/mL, once per week from day 48–103). A detailed timeline is illustrated in Figure S1. Mice were euthanized by cervical dislocation 24 h after the final administration. Serum was collected to measure IgE levels using an ELISA kit. Mouse heads were resected from the second palatal ridge to the first maxillary molar, and specimens were fixed in 4% paraformaldehyde for 48 h.

#### Analysis of tandem mass tag (TMT)-based quantitative proteomics

Quantitative proteomics analysis was performed by GeneChem (Shanghai, China). Briefly, total proteins were extracted from nasal mucosa tissue samples and quantified using the BCA method. Equal amounts of protein were reduced with dithiothreitol, alkylated with iodoacetamide, and subjected to tryptic digestion using the filter-aided sample preparation (FASP) method to obtain peptide mixtures. Peptides from each sample were labeled with specific TMT reagents and subsequently pooled in equal amounts. To reduce sample complexity, the pooled peptides were fractionated by high-pH reverse-phase high-performance liquid chromatography and combined into 10 fractions. Each fraction was separated online using a nano-liquid chromatography system with an acetonitrile gradient containing 0.1% formic acid. Mass spectrometric analysis was performed on a Q Exactive HF-X mass spectrometer (Thermo Fisher Scientific) in data-dependent acquisition (DDA) mode. The full scan resolution was set to 120,000, and the top 10 most intense precursor ions were selected for higher-energy collisional dissociation (HCD) with an MS2 resolution of 45,000. Raw data were processed using the Proteome Discoverer software platform (version 2.4). Protein identification and quantification were conducted by searching the UniProt Homo sapiens database (version 20231008) via the MASCOT search engine (version 2.6). Search parameters included: trypsin digestion with up to 2 missed cleavages, precursor ion mass tolerance of 10 ppm, fragment ion mass tolerance of 0.05 Da, fixed modification of carbamidomethylation on cysteine, and variable modifications of methionine oxidation and protein N-terminal acetylation. The false discovery rate (FDR) for proteins and peptides was strictly controlled below 1%. Proteins with a fold change >1.5 (or 2.0) and a *p*-value <0.05 were considered differentially expressed. Bioinformatics analyses, including Gene Ontology (GO) functional annotation and KEGG pathway enrichment analysis, were performed on these differentially expressed proteins; GO terms and KEGG pathways with a *p*-value <0.05 were considered statistically significant. All mass spectrometry proteomics data have been deposited to the ProteomeXchange Consortium via the PRIDE partner repository (dataset identifier: PXD079333).

### Quantification and statistical analysis

Data are presented as mean ± SD from at least three independent biological replicates (*n* ≥ 3). The exact value of n and what it represents (e.g., number of animals or independent cell culture replicates) are indicated in the respective figure legends. All statistical analyses were performed using GraphPad Prism 10.0. Normality and homogeneity of variances were confirmed before applying parametric tests. For comparisons between two groups, an unpaired Student’s *t* test was used; for comparisons across multiple groups, one-way ANOVA followed by Tukey’s multiple comparisons test was applied. Statistical significance was defined as: ∗*p* < 0.05, ∗∗*p* < 0.01, ∗∗∗*p* < 0.001.

### Additional resources

This study did not involve a clinical trial and is not registered in any clinical registry repository.
