# Supplementary material for: DNAJB9 promotes epithelial-mesenchymal transition in the nasal epithelium of chronic rhinosinusitis by enhancing TRIM22-mediated IκBα degradation
Source: iScience. 2026 Jul 16;29(8):116681. doi: 10.1016/j.isci.2026.116681 (PMC13400852; doi:10.1016/j.isci.2026.116681)
Supplement: Document S1. Figures S1–S3 and Tables S1 and S2 [file mmc1.pdf]

iScience, Volume *29*

Supplemental information

DNAJB9 promotes epithelial-mesenchymal transition in the nasal epithelium of chronic rhinosinusitis by enhancing TRIM22-mediated IκBα degradation

Zhiqiang Zhang, Xinyu Huang, Junhao Tu, Mengyuan Liu, Ying Wu, Xu Zhang, Yun Zhang, and Chunping Yang

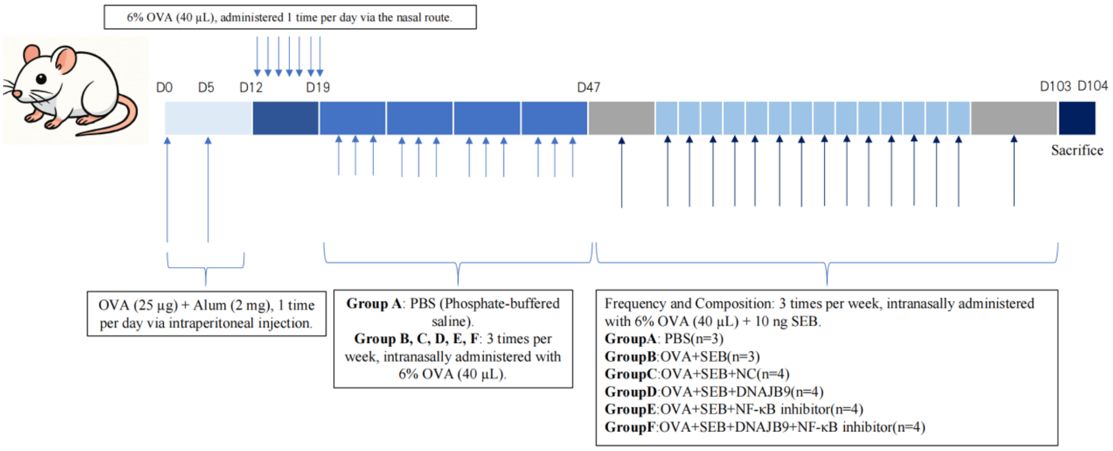

Figure S1. Timeline diagram of the construction and experimental grouping of the CRSwNP mouse model, Related to Figure 1 and Figure 3. BALB/c mice were sensitized by intraperitoneal injection of ovalbumin (OVA, 25 μg) and aluminum hydroxide (2 mg) on day 0 and day 5. From day 12 to day 103, 6% OVA (40 μL) was instilled intranasally to induce and maintain inflammation. From day 48 onwards, mice received intranasal instillation of a combination of OVA and Staphylococcal enterotoxin B (SEB, 10 ng) three times per week to promote nasal polyp formation. Mice were randomly divided into six groups (n = 3-4 per group): Group A (PBS control), Group B (OVA+SEB model), Group C (OVA+SEB + negative control), Group D (OVA+SEB + DNAJB9 overexpression), Group E (OVA+SEB + DBS inhibitor), Group F (OVA+SEB + DNAJB9 overexpression + NF-κB inhibitor). All mice were sacrificed on day 104 for analysis.

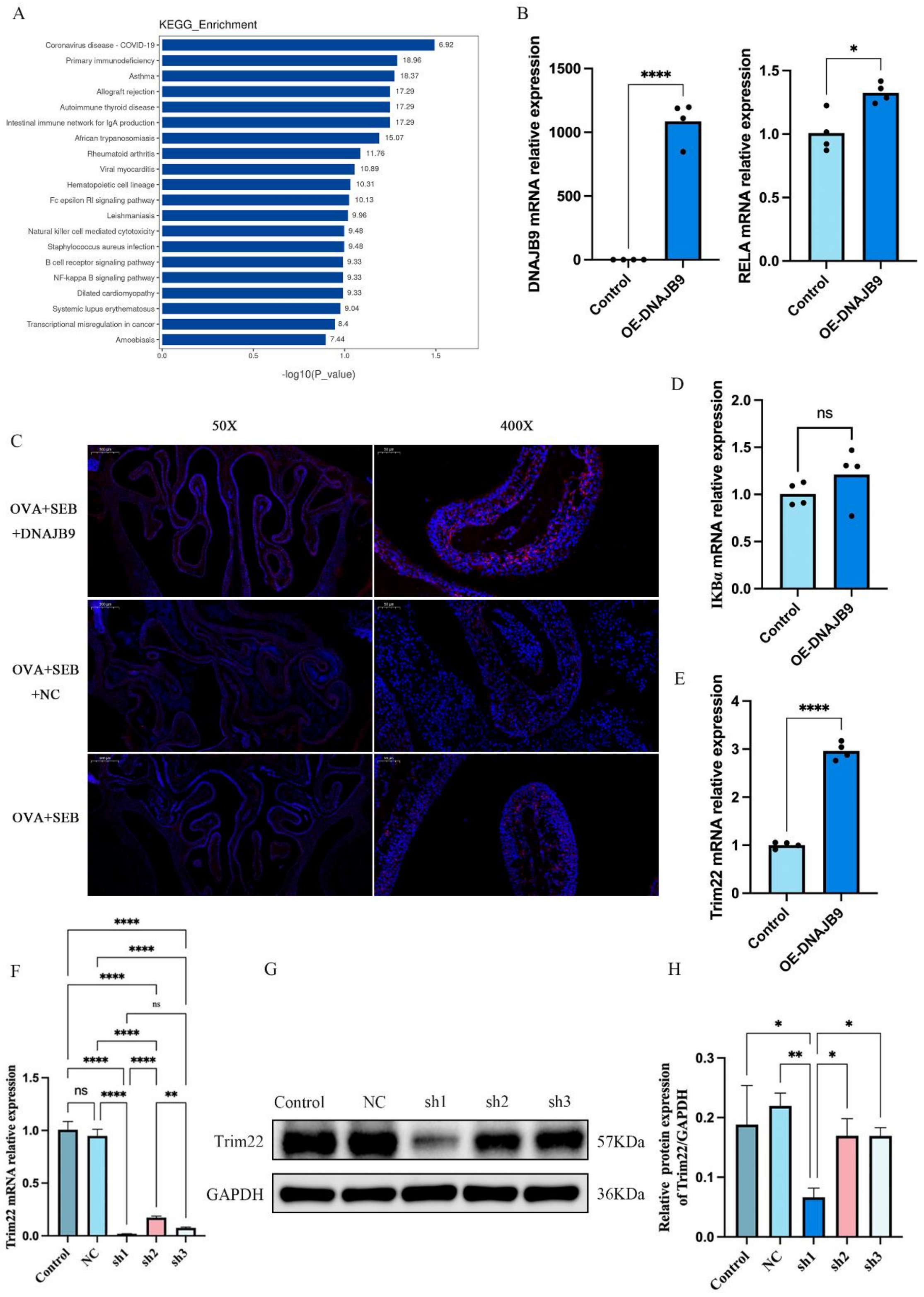

Figure S2. TMT quantitative proteomics analysis and validation of DNAJB9 stable knockdown efficiency, Related to Figure 1 and Figure 2. A Based on TMT quantitative proteomics data, proteins co-up-regulated among three groups of healthy

controls (HC), chronic sinusitis without nasal polyps (CRSsNP), and chronic sinusitis with nasal polyps (CRSwNP) were shown by Wayne diagram. B Based on quantitative proteomics data, DNAJB9 expression levels were shown in chronic sinusitis with nasal polyps (CRSwNP) compared with chronic sinusitis without nasal polyps (CRSsNP) and healthy controls (HC). C HE staining showed the histomorphology of the nasal mucosa in the control and nasal polyp model mice. Low-power row (magnification: 50×; Scale bar = 500 µm) showed the overall structure of the nasal mucosa; high-power row (magnification: 400×; Scale bar = 50 µm) clearly showed pathological changes such as mucosal epithelial edema, inflammatory cell infiltration and polypoid hyperplasia in the model group. D Quantitative analysis of the relative mRNA expression of DNAJB9. E Representative Western Blot plots following stable knockdown of DNAJB9 by shRNA in BEAS-2B cells. F Quantitative analysis of DNAJB9 protein levels. Data are presented as mean $\pm$ standard deviation. For Panel B, data are presented as box plots showing the median and interquartile range. For Panels D and F, data are presented as mean $\pm$ standard deviation (SD). Statistical significance was determined using the unpaired two-tailed Student's t-test for Panel B, and one-way ANOVA followed by Tukey's post-hoc test for Panels D and F. *P < 0.05, P < 0.01, ***P < 0.001.

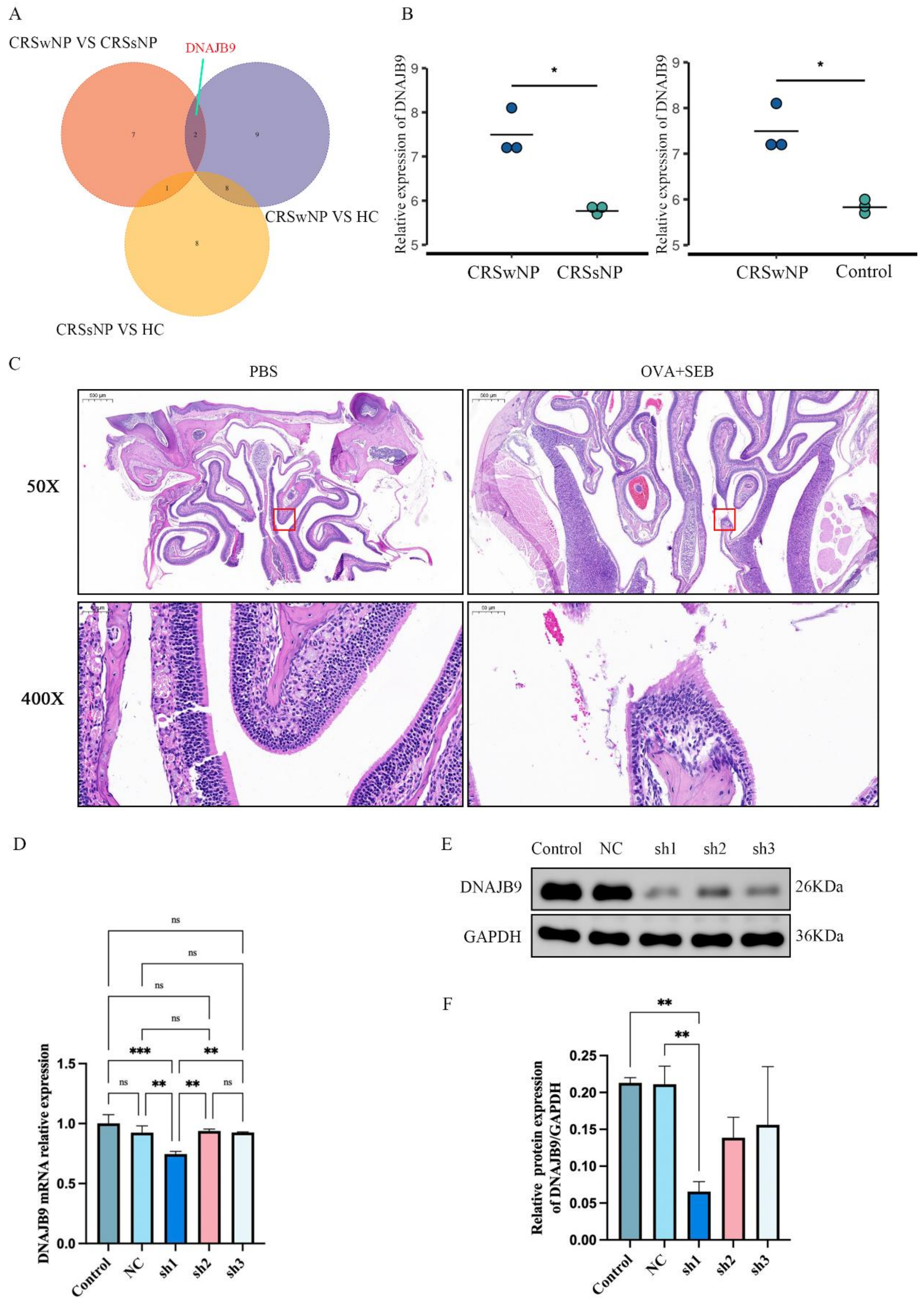

Figure S3. KEGG enrichment, localized expression, and verification of Trim22 knockdown, Related to Figure 3, Figure 4, and Figure 5. A KEGG pathway enrichment analysis of differentially expressed proteins in the CRSwNP group was

performed based on TMT quantitative proteomics data. Representative Western Blot plots following stable knockdown of DNAJB9 by shRNA in BEAS-2B cells. B Quantitative analysis of the relative mRNA expression of DNAJB9 and RELA. C Representative immunofluorescent staining pictures of nasal mucosa tissues from mice in each group. Left column (magnification: 50×; Scale bar = 500 μm) shows the overall structure of the tissue; right column (magnification: 400×; Scale bar = 50 μm) clearly shows the expression and localization of DNAJB9 (red) in nasal mucosal epithelial cells. Nuclei were stained with DAPI (blue). D Quantitative analysis of the relative mRNA expression of IκBα. E Quantitative analysis of the relative mRNA expression of Trim22. F Quantitative analysis of the relative mRNA expression of Trim22. G Representative Western Blot plots following stable knockdown of Trim22 by shRNA in BEAS-2B cells. F Quantitative analysis of Trim22 protein levels. Data are presented as mean ± standard deviation. For all panels presenting pooled quantitative data (B, D, E, F, and H), data are presented as mean ± standard deviation (SD). Statistical significance was determined using the unpaired two-tailed Student's t-test for Panels B, D, and E, and one-way ANOVA followed by Tukey's post-hoc test for Panels F and H. $^{*}P < 0.05$, $^{**}P < 0.01$, $^{***}P < 0.001$.

Table S1 qPCR primers used in this study.

Gene	Forward primer(5′ to 3′)	Reverse primer(5′ to 3′)
GAPDH	GTCTCCTCTGACTTCAACAGCG	ACCACCCTGTTGCTGTAGCCAA
β-actin	TGGCACCCAGCACAATGAA	CTAAGTCATAGTCCGCCTAGAAGCA
Ecadherin	GCCTCCTGAAAAGAGAGTGGAAG	TGGCAGTGTCTCTCCAAATCCG
Ncadherin	CCTCCAGAGTTTACTGCCATGAC	GTAGGATCTCCGCCACTGATTC
Vimentin	GAGGAAGCCGAAAACACCCT	TTGCGTTCAAGGTCAAGACG
DNAJB9	AGGACAAAGAGGTAGTGGAAGT	CCTGGCGTGTCTGGAAATGA
Trim22	GCTGTGCCTCCCTGTCGTATTG	ATGAGTGCTCCGTGGTTTGTGAC
NFKBIA	CCCGCACCTCCACTCCATCC	AGCATTGACATCAGCACCCAAG
RELA	TGTGAAGAAGCGGGACCTGGAG	AAGCAGAGCCGCACAGCATTC

Table S2 Antibody used in this study.

Antigen	Species	Product No, Manufacturer	Dilution
**DNAJB9**	Rabbit	13157-1-AP，Proteintech	IHC: 1:100, Western blot: 1:2000, IF: 1:100
**Vimentin**	Rabbit	ET1610-39, Huabio	IHC: 1:200, Western blot: 1:10000
**E-cadherin**	Rabbit	20874-1-AP，Proteintech	IHC: 1:200, Western blot: 1:10000
**N-cadherin**	Rabbit	ER0503, Huabio	IHC: 1:200, Western blot: 1:2000
**NF-κB, p65**	Rabbit	10745-1-AP，Proteintech	Western blot: 1:1000
**p NF-κB, pP65**	Rabbit	82335-1-RR, Proteintech	Western blot: 1:1000
**Ikbα**	Rabbit	10268-1-AP, Proteintech	Western blot: 1:1000
**Ikbα**	Mouse	15595, CST	IF: 1:200
**Trim22**	Rabbit	13744-1-AP, Proteintech	Western blot: 1:1000
**Flag**	Rabbit	20543-1-AP, Proteintech	Western blot: 1:10000, 1 μg for IP
**Flag**	Mouse	66008-4-Ig, Proteintech	Western blot: 1:10000, 1 μg for IP
**MYC**	Rabbit	16286-1-AP, Proteintech	Western blot: 1:10000, 1 μg for IP
**MYC**	Mouse	60003-2-Ig, Proteintech	Western blot: 1:10000, 1 μg for IP
**GFP**	Rabbit	50430-2-AP, Proteintech	Western blot: 1:10000, 1 μg for IP
**GFP**	Mouse	66002-1-Ig, Proteintech	Western blot: 1:10000, 1 μg for IP
**HA**	Rabbit	51064-2-AP, Proteintech	Western blot: 1:10000, 1 μg for IP
**HA**	Mouse	66006-2-Ig, Proteintech	Western blot: 1:10000, 1 μg for IP
**Ubiquitin**	Rabbit	10201-2-AP, Proteintech	Western blot: 1:1000
**GAPDH**	Rabbit	10494-1-AP, Proteintech	Western blot: 1:10000
**β-Actin**	Rabbit	R1207-1, Huabio	Western blot: 1:10000
**Anti-IgG**	Rabbit	SA00001-2, Proteintech	Western blot: 1:10000
**Anti-IgG**	Mouse	SA00001-1, Proteintech	Western blot: 1:10000
